# Supplementary material for: Content-rich biological network constructed by mining PubMed abstracts
Source: BMC Bioinformatics. 2004 Oct 8;5:147. doi: 10.1186/1471-2105-5-147 (PMC528731; doi:10.1186/1471-2105-5-147)
Supplement: Additional File 2 — The original results of the above study (non-essential files are deleted to keep the file size under the limit set by BMC bioinformatics). [file 1471-2105-5-147-S2.bz2 › chilibotAdditionalFile2/dip10/663ID9450761E11056/html/NFKB1_RELA.html]

 


 **NFKB1** and **RELA** 
  
Found 53 abstracts in PubMed, retrieved 10.  
 

 What does Google say? 
 PDF only 
| .edu only 

---

**Interactive relationship** (e.g. stimulation, inhibition, etc)

**Non-interactive relationship** (e.g. studied together, co-existance, homology, etc.)

- To investigate whether transcription factors of the NF kappa B family could play a role in early mammalian development, we have analyzed the expression of  **nfkb1** , nfkb2, c Rel,  **RelA** , RelB, and bcl 3 from 6.5 to 10.5 day mouse embryo implantation sites.  Ref: 10402669 Dev Genet, 1999
- LY294002, PI3K DN, or AKT DN inhibited NFkB  [ **NFKB1** ]  activation, p65  [ **RELA** ]  transactivation, and cyclooxygenase 2 COX 2 expression induced by lauric acid or constitutively active CA TLR4.  Ref: 12865424 J Biol ChemJ Biol Chem, 1999
- MS association was excluded for the  **NFKB1**  and NFKB3  [ **RELA** ]  genes, which show remarkably low degrees of polymorphism.  Ref: 12058256 Genes Immun, 2002
- The gel supershift assay with  **Nfkb1** ,  **Rela**  and or Rel antibodies revealed that the specific molecular forms of NF kappaB activated by radiation in the spleen were  **Nfkb1**  homodimers and  **Nfkb1**   **Rela**  heterodimers.  Ref: 10360790 Radiat Res, 1999
- Using antibodies, we demonstrated that these complexes contain NFkappaB subunits  **NFkB1** , NFkB2,  **RelA**  and c Rel.  Ref: 10773315 Virus Res, 2000
- In bone marrow, an NF kappaB like binding factor was induced that may be  **Nfkb1**   **Rela**  and Rel  **Rela**  like heterodimers, but it exhibited a higher mobility than  **Nfkb1**  homodimers.  Ref: 10360790 Radiat Res, 1999
